# Anticholinergic medication index: advancing safer prescribing for older adults

**DOI:** 10.1136/bmjmed-2026-003197

**Published:** 2026-11-04

**Authors:** Prasad S Nishtala, Gulnaz Gill

**Affiliations:** 1 Life Sciences, University of Bath, Bath, UK; 2 Patient and Public Involvement Contributor, Bristol, UK

**Keywords:** Health policy, Epidemiology, Medical informatics, Geriatrics, Primary health care

The new anticholinergic medication index helps integrate evidence based risk assessment into clinical decision making to prevent medication harm in older adults

Older adults (≥65 years) frequently live with coexisting long term conditions^1^ and regularly take multiple drugs to manage them, making this group particularly susceptible to medication related harm.^2,3^ Some of the most important contributors are medicines with anticholinergic properties,^4^ which have consistently been associated with delirium, falls, cognitive impairment, functional decline, and admission to hospital.^5–7^ However, despite widespread recognition of these risks, clinicians have lacked a practical, evidence based tool that can be seamlessly integrated into routine electronic health records to identify patients who may benefit most from medication review.

Best and colleagues (doi: 10.1136/bmjmed-2026-002835) describe the development and external validation of the anticholinergic medication index (ACMI), a novel approach that moves beyond traditional expert consensus based anticholinergic burden scales.^8^ The ACMI derives drug weights from a clinical prediction model for delirium and falls developed using routinely collected healthcare data from almost 750 000 older adults in England and Wales. The ACMI generates a numerical score ranging from 0 to 100, reflecting cumulative anticholinergic medication burden rather than a percentage risk. A patient who is prescribed no anticholinergic drugs would have an ACMI score of 0, whereas a patient who was hypothetically prescribed all 88 anticholinergic drugs in the index would have the theoretical maximum ACMI score of 100.

The concept of anticholinergic burden is not new: over the past two decades, numerous tools (including the anticholinergic cognitive burden scale^9^ and anticholinergic drug scale^10^) have attempted to quantify exposure to anticholinergic medicines. These tools have contributed substantially to research and have helped raise awareness among clinicians. However, these tools also share important limitations. Anticholinergic scores are typically derived through expert consensus; different scales often assign different scores to the same drug, and most of these tools are not well integrated into routine clinical practice.^11,12^ Consequently, despite strong evidence linking anticholinergic use with adverse outcomes, these tools have had limited influence on everyday prescribing decisions.^13^ The drug burden index quantifies cumulative exposure to anticholinergic and sedative drugs by incorporating dose. Despite limitations of the simple linear additive pharmacological model used in the drug burden index, this model is widely used in research and clinical practice, for example, in electronic drug reviews in Australia.^14,15^

The ACMI resolves some of these shortcomings. From the perspective of older people and their carers, even a single fall or episode of delirium can have consequences that extend well beyond the acute event, such as loss of confidence, reduced independence, and increased reliance on family members and health and social care services.^8^ By deriving anticholinergic drug weights directly from associations with hospital admissions resulting from delirium or serious falls, Best and colleagues have developed a score explicitly linked to outcomes that matter most to older people. This outcome based approach strengthens the clinical relevance of the score and reinforces the importance of identifying and reducing drug related harm that can profoundly affect quality of life and functional independence.^16^

The linked study also illustrates the growing role of prediction models in supporting clinical decision making. Importantly, however, prediction tools should support, not replace, clinical judgement. The ACMI estimates risk, but does not establish whether drugs with anticholinergic properties directly caused an delirium or a fall at the individual level.^8^ Many medicines with anticholinergic properties remain clinically appropriate and may provide substantial benefits. Likewise, identification of a high ACMI score should prompt thoughtful drug review rather than automatic deprescribing.^17^

The authors of the linked study explain that the ACMI is not intended to be used in isolation, rather that the index should be considered with a separately estimated one year risk of delirium or falls in a given patient.^8^ Looking at these risks together may help clinicians to identify patients who might benefit from a drug review and, where appropriate, deprescribing. Nevertheless, several questions remain. Although external validation demonstrated good discrimination, calibration was less consistent, highlighting the need for further evaluation in different healthcare settings and populations. The model also simplifies drug use by treating drugs as binary variables, rather than incorporating dose or patient adherence. While this pragmatic calculation of anticholinergic scores improves feasibility within electronic health records, pharmacological precision is inevitably lost. Future iterations may benefit from incorporating richer prescribing information as digital infrastructure evolves. Similarly, because drugs and prescribing patterns continue to change over time, the index must be updated to maintain accuracy.

In an era where healthcare systems increasingly seek scalable approaches to drug optimisation, implementation may prove as important as methodological innovation. Perhaps the most important unanswered question is whether implementation of the ACMI improves patient outcomes. The logical next step is implementation research to determine whether structured drug reviews that are guided by the ACMI and actively involve patients (as well as, where appropriate, their carers) in shared decision making can reduce instances of falls and delirium, lower use of healthcare services, and improve quality of life.^18^ Such studies should also assess clinician acceptability, workflow integration, equity, and cost effectiveness.

As populations age and use of multiple medicines is already recognised as a norm rather than the exception, clinicians need decision support tools for drug optimisation that are evidence based, practical, and easily incorporated into routine care.^19^ Best and colleagues have taken an important step towards that goal by developing a clinically grounded, electronic measure of anticholinergic burden. The challenge now is to demonstrate that integrating such tools into routine practice translates into better prescribing decisions and, ultimately, better outcomes for older people.
